# Determinants for a low dose of alteplase and its relationship to a lower intracerebral bleeding risk in acute ischemic stroke

**DOI:** 10.7150/ijms.76105

**Published:** 2022-10-03

**Authors:** Jie Chen, Hangfeng Li, Hanhan Lei, Shuangfang Fang, Qilin Yuan, Yangui Chen, Dongping Chen, Ronghua Chen, Yixian Zhang, Jin Wei, Guangliang Chen, Zhiting Chen, Nan Liu, Hou-wei Du

**Affiliations:** 1 Department of Neurology, Fujian Provincial Hospital South Branch, Fuzhou, China.; 2 Department of Neurology, Longyan First Hospital of Fujian Medical University, Longyan, Fujian, China.; 3 Department of Neurology, Fujian Medical University Union Hospital, Fuzhou, China.; 4 Institute of Clinical Neurology, Fujian Medical University, Fuzhou, China.; 5 Department of Rehabilitation, Fujian Medical University Union Hospital, Fuzhou, China.; 6 Department of Radiology, Fujian Medical University Union Hospital, Fuzhou, Fujian, China.

**Keywords:** acute ischemic stroke, low-dose, alteplase, symptomatic intracranial hemorrhage

## Abstract

**Background:** Factors for the utilization of intravenous thrombolysis with a low-dose of alteplase (0.6mg/kg) and whether the low-dose of alteplase could reduce the risk of intracerebral bleeding in acute ischemic stroke (AIS) remains uncertain.

**Aims:** We aimed to investigate determinants for the utilization of intravenous thrombolysis with a low-dose of alteplase. We further assessed the association between the low-dose of alteplase and the intracerebral bleeding risk in AIS patients.

**Method:** We included AIS patients who received intravenous thrombolysis using alteplase in this multicenter retrospective observational study. We investigated the association between baseline characteristics and the utilization of a low-dose of alteplase to identify determinants. We assessed the association of the low-dose of alteplase with the risk of symptomatic intracranial hemorrhage (sICH) using a multivariable logistic regression model. We further compared the rate of sICH and any ICH in patients in the low-dose group to those in the standard-dose group, using propensity score-matching data.

**Results:** A total of 506 AIS patients were included in this study. The mean age was 67 (interquartile range [IQR] 59-75), and 178 (35.2%) were women. A total of 96 patients were treated with the low-dose. Age (adjusted odds ratio [OR] 1.02, 95% confidence interval [CI] 1.00 -1.04, p = 0.042), having a previous ischemic stroke (adjusted OR 2.01, 95%CI 1.11 - 3.64 p = 0.021) and increasing baseline systolic blood pressure (adjusted OR 1.12, 95%CI 1.00 - 1.26, p = 0.049) were determinants for the utilization of the low-dose. Multivariable logistic regression analysis showed that the low-dose was significantly associated with a reduced risk of sICH (adjusted OR 0.13, 95%CI 0.03 - 0.62, p = 0.01). Propensity score analysis showed that the rate of sICH was significantly lower in the low-dose group compared to standard-dose group (2 [2.3%] vs 10 [11.4%], p = 0.032). There was no significant difference in the rate of any ICH between two groups (14 [15.9%] vs 18 [20.5%], p = 0.434).

**Conclusions:** Patients with increasing age, a higher baseline systolic blood pressure, and previous ischemic stroke were at a higher odd of receiving a low-dose of alteplase. The low-dose was associated with a lower risk of developing symptomatic intracranial hemorrhage.

## Introduction

Current stroke guidelines 1-3 recommend intravenous thrombolysis (IVT) using the recombinant tissue plasminogen activator (r-tPA) at a standard-dose (0.9mg/kg, alteplase) for patients with acute ischemic stroke (AIS). However, substantial concerns have been raised regarding the risk of adverse effects caused by r-tPA, of which the symptomatic intracranial hemorrhage (sICH) associates with poor clinical outcomes 4-6. Whether a low-dose (0.6mg/kg, alteplase) of r-tPA could reduce the risk of intracerebral bleeding with similar efficacy as that of the standard-dose has long been debated. The Enhanced Control of Hypertension And Thrombolysis Stroke Study (ENCHANTED) showed that patients in the low-dose group experienced fewer sICH events than in the standard-dose group, although the low-dose was not non-inferior to standard-dose in reducing death and disability when administered within 4.5 hours of stroke onset 7. The Japan stroke guideline recommends IVT using the low-dose of r-tPA 8. Recently, several meta-analyses investigated the association between the low-dose and the risk of sICH, yielding inconsistent results 9,10. The present study aimed to explore the potential determinants for the utilization of the low-dose of r-tPA, and whether the low-dose was associated with a lower intracerebral bleeding risk in this retrospective observational study.

## Methods

### Study design, participants, and setting

This is a retrospective, multi-center observational study conducted in stroke centers of three tertiary teaching hospitals in Southeast China. We included eligible adult (18 years or older) AIS patients who were treated with IVT using r-tPA within 4.5 hours after symptom onset. We applied the following exclusion criteria: 1. Patients with missing data regarding r-tPA dose; 2. Those who received arterial thrombolytic treatment; 3. Those who underwent interrupted IVT because of rapid neurological function improvement or severe side effects; 4. Those with missing data regarding sICH information. This study was approved by the local ethics committees at each center. Written informed consents were waived due to the nature of our retrospective study using routine anonymous data.

### Data extraction

Two authors (J.C. and H.L.) reviewed the electronic medical records using a digital database, and extracted data regarding base demographics and clinical characteristics using standard digital collection sheets.

### Intervention and Evaluation

The low-dose of alteplase (Boehringer Ingelheim Pharma GmbH) was defined as the dose of 0.6mg/kg body weight, and the standard-dose was defined as the dose of 0.9mg/kg body weight, based on current stroke guidelines 3,8. A single dose of 0.9 mg/kg body weight (not exceeding 90 mg) or 0.6 mg/kg body weight (not exceeding 60 mg) was administered intravenously, with 10% (for 0.9mg/kg) or 15% (for 0.6 mg/kg) given as a bolus, followed by continuous infusion of the remainder within one hour. Stroke severity (presenting deficit severity) was assessed using the National Institutes of Health Stroke Scale (NIHSS) score by qualified clinicians before and after IVT treatment 11. Functional outcome at discharge was assessed using a modified Rankin Scale (mRS, ranging from 0 to 6, with higher scores indicating greater disability) 12.

### Outcome measures

Our primary outcome is symptomatic intracranial hemorrhage (sICH), defined as computed tomography (CT) evidence of new ICH with documented objective evidence of neurological decline or an increase of four points from the most recent NIHSS score, based on the European Cooperative Acute Stroke Study (ECASS-II) criteria 13. Another safety outcome is any ICH, defined as CT-visible intracranial hemorrhage with and without evidence of neurological decline 13. Additional outcomes include discharge NIHSS score, proportion of good functional outcome defined as a mRS score of 0 to 3 12, and in-hospital mortality.

### Statistics

Continuous variables were expressed as mean with standard deviation (SD) and median [interquartile range (IQR)] for those with normal and skewed distributions, respectively. Categorical variables were expressed as frequency with percentage. Between groups differences in demographic and clinical characteristics were calculated with t-test (for continuous variable if normally distributed) or Mann-Whitney test (if skewed distributions). Categorical variables were compared by Chi-square test or fisher's exact test, as appropriate. A multivariable logistic regression model was established to assess the association between baseline characteristics and the utilization of the low-dose. We calculated the unadjusted, and age- sex- adjusted odds ratio (OR) of sICH events with the low-dose of alteplase. The relationship between the low- dose and sICH risk was further assessed using multivariable logistic regression analysis by introducing those with a p < 0.1 in univariable analysis in addition to age and sex. To generate a comparable data set, we calculated a propensity score to estimate the individual probability of a patient receiving the low-dose of alteplase. Four variables were used as covariates (age, baseline NIHSS score, baseline systolic blood pressure (SBP), and history of a previous stroke). All statistical analyses were conducted using the SPSS 25.0 for windows (IBM). A p-value <0.05 was considered statistically significant.

## Results

**Figure 1:**
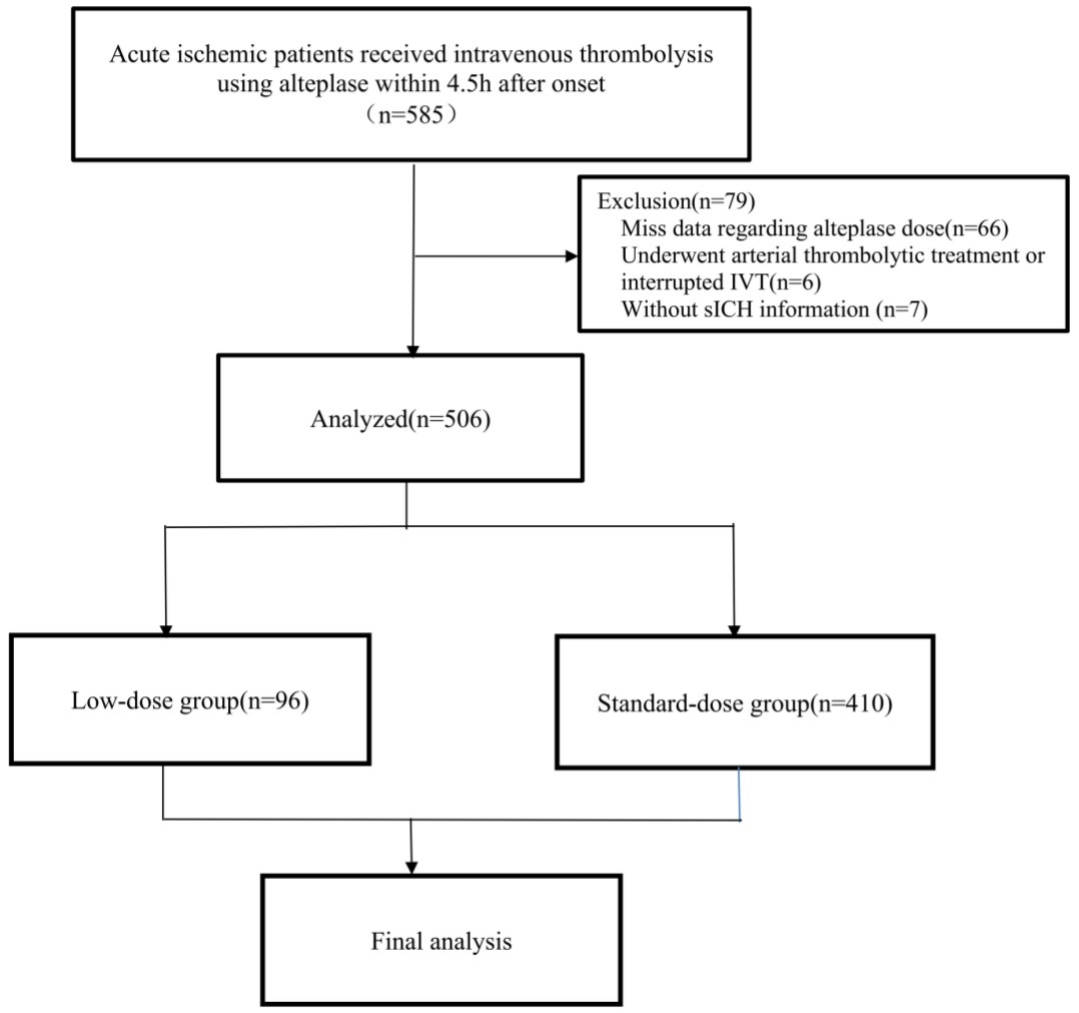
Flowchart of the study. Abbreviations: AIS, acute ischemic stroke; IVT, intravenous thrombolysis; sICH, symptomatic intracerebral hemorrhage.

Between 2017 and 2021, a total of 585 AIS patients received IVT using r-tPA within 4.5 h after onset in this retrospective study. After excluding 79 patients (66 with missing data regarding r-tPA dose, 6 underwent arterial thrombolytic treatment or interrupted IVT, and 7 without sICH information), 506 patients were included in the final analysis (**Figure 1**). Ninety-six [19.0%] patients received a low dose of r-tPA. There were no significant differences in age (67 [IQR 59-75] vs 64 [58-74], p = 0.437), proportion of female sex (178 [35.2%] vs 29 [36.7%], p = 0.791), history of hypertension (342 [67.6%] vs 46 [58.2%], p = 0.102), diabetes (112 [22.1%] vs 14 [17.7%], p = 0.375), atrial fibrillation (162 [32.0%] vs 26 [32.9%], p = 0.874), previous ischemic stroke (67 [13.2%] vs 7 [8.9%], p = 0.276), and baseline NIHSS score (8 [IQR 4-15] vs 6 [IQR 3-11], p = 0.153) between those included and excluded in the final analysis.

**Table 1** shows the differences in demographics and clinical characteristics among patients in the low-dose and standard-dose groups. Patients in the low-dose group were more likely to be older (median age, 69 [IQR 63-79] vs 67 [58-74], p = 0.011), had a previous ischemic stroke (20 [20.8%] vs 47 [11.5%], p = 0.015), a higher median baseline SBP (155mmHg [IQR 132-168] vs 147 [134 - 160], p = 0.017), and a higher baseline NIHSS score (8 [4-12] vs 6 [3-11], p=0.022). Regarding radiological findings, patients in the low-dose group were more likely to have hyperdense middle cerebral artery (MCA) sign on baseline non-contrast CT (12 [12.9%] vs 14 [3.5%], p < 0.001, data available in 490 patients).

**Table 2** shows the association between baseline characteristics and the utilization of the low-dose, using a multivariable logistic regression analysis. The results showed that age (adjusted OR 1.02, 95%CI 1.00 - 1.04, p = 0.042), having a previous ischemic stroke (adjusted OR 2.01, 95%CI 1.11 - 3.64, p = 0.021) and having a higher baseline SBP (per-10mmHg increase, adjusted OR 1.12 95%CI 1.00 - 1.26, p = 0.049) were determinants for the utilization of the low-dose. There was a borderline association between baseline NIHSS score and the utilization of the low-dose (per-point increase, adjusted OR 1.04, 95%CI 1.00-1.08, p = 0.057).

A total of 34 (6.7%) sICH and 89 (17.6%) any ICH events were observed. Fatal sICH occurred in three patients who received the standard-dose alteplase, compared to none in the low-dose group. The rate of sICH was lower in the low-dose group compared to the standard-dose group, although only showing a trend towards the statistical significance (2[2.1%] vs 32 [7.8%], p = 0.074). The rate of any ICH was not significantly different between the low-dose and the standard-dose group (15 [15.6%] vs 74 [18.0%], p = 0.574). Patients in the low-dose and standard-dose groups did not differ in discharge NIHSS score (2 1-5 vs 3 1-6, p = 0.931), proportion of a discharge mRS score of 0-3 (74 [77.1%] vs 296 [72.2%], p = 0.331) and in-hospital mortality (0 vs 12 [2.9%], p = 0.135).

**Table 3** summarizes the differences in baseline demographics and clinical characteristics between patients with and without sICH. Patients with sICH were older (median age, 73 [IQR 68-80] vs 67 [58-74], p < 0.001), more likely to have atrial fibrillation (25 [73.5%] vs 137 [29.0%], p < 0.001), diabetes (12 [35.3%] vs 100 [21.2%], p = 0.056), and a higher baseline NIHSS score (14 [IQR 9.5-17.5] vs 5 [3-11], p < 0.001). A lower proportion of hyperlipidemia were observed in patients with sICH compared to those without (7 [20.6%] vs 176 [37.3%], p = 0.05). Multivariable logistic regression analysis showed that the low-dose was significantly associated with a reduced risk of sICH (adjusted OR 0.13, 95%CI 0.03 - 0.62, p = 0.010, **Table 4**).

**Table 5** shows that there were no significant differences in baseline demographics and clinical characteristics between patients who in the low-dose group and standard-dose group derived from a propensity score-matching cohort. The propensity score-matching analysis (88 in the low-dose group vs 88 in the standard-dose group) showed that the rate of sICH was significantly lower of the low-dose group compared to that of the standard-dose group (2 [2.3%] vs 10 [11.4%], p = 0.032). There was no significant difference in the rate of any ICH (14 (15.9%) vs 18 (20.5%), p = 0. 434), discharge NIHSS score (2 1-5 vs 2 1-7, p = 0.732), proportion of a mRS score of 0-3 (61 [69.3%] vs 56 [63.6%], p = 0.425), and in-hospital mortality (0 vs 3 [3.3%], p = 0.246) between two groups.

## Discussion

The present study showed that AIS patients with increasing age, a higher baseline SBP and previous stroke were at a higher odd of receiving the low-dose of alteplase. Moreover, the low-dose of alteplase was associated with a lower risk of developing symptomatic intracranial hemorrhage.

Previous studies showed that the utilization of the low-dose was mostly clinical relevant, including the prior use of antiplatelet treatments, antecedents of previous significant hemorrhage, or conditions that contraindicated the use of r-tPA 13. Our data showed that patients who received a low dose of r-tPA were older (69 [63-79] vs 67 [58-74]), had a higher baseline SBP (155mmHg [132 - 168] vs 147 [134 - 160]), and a higher baseline NIHSS score (8 [4-12] vs 6 [3-11]). Our findings were supported by data from several multicenter, nationwide stroke registry-based observational studies 15-17. The US stroke guideline does not limit advanced age 1. However, the ECASS trials excluded those aged 80 years or older 13. Observational data from the Safe Implementation of Treatments in Stroke (SITS) International Stroke Thrombolysis Register demonstrated the association between high baseline SBP and the risk of sICH 5. Stroke severity assessed using the NIHSS score was the most relevant for an increased risk of sICH in AIS population. A meta-analysis of multiple randomized controlled trials showed that the risk of fatal sICH increased significantly with baseline NIHSS score in AIS patients who received intravenous alteplase 18. However, IVT improved the overall likelihood of a good functional outcome at 3-6 months, irrespective of age or stroke severity 18. Taken together, the low-dose of r-tPA would be favored when patients are thought to be at high bleeding risk, such as in older patients, or those with a higher baseline SBP and presenting a severe stroke.

In our cohort, the proportion of previous stroke was higher in the low-dose group compared to the standard-dose group (20 [20.8%] vs 47 [11.5%]). Patients with a history of ischemic stroke were excluded from most randomized controlled trials 12, 19, 20. Consistent with our findings, an individual participant data of observational studies showed that the percentage of previous stroke was higher in patients who received a low-dose of alteplase compared to a standard-dose (1149 [20.6%] vs 657 [18.5%]), although the difference was not statistically significant 15. There are several possible explanations. First, having a history of stroke within 90 days before the index stroke event is listed as a contradiction for IVT, based on the China stroke guideline 3. However, some clinicians prefer to prescribe IVT to those with a low risk of intracerebral bleeding, based on their best clinical practice. A retrospective study 21 showed that having a history of previous stroke in less than three months did not translate to a worse outcome than those with first-ever stroke (OR: 1.62, 0.54 - 4.83). However, the wide 95% CI does not exclude the potential risk of intracerebral bleeding. Whether the low-dose of alteplase could reduce the risk of cerebral bleeding in patients with recurrent ischemic stroke remains unresolved and needs to be investigated in future large sample-sized studies.

Our univariate analysis showed that the low-dose was non-statistically significantly different among patients with and without sICH (although there was a trend towards the statistical significance). However, our multivariate analysis showed that the low-dose was associated with a lower risk of sICH (adjusted OR 0.13, 95%CI 0.03 - 0.62, p = 0.01). One potential explanation is that patients in the low-dose group were older, had a higher baseline SBP, and presented a more severe stroke, all of which are risk factors for sICH 5,22. Therefore, the between group differences may be underestimated. Our propensity score matching data confirmed the association between the low-dose alteplase and a reduced risk of sICH, after balancing these factors. Notably, the low-dose and standard-dose did not differ in discharge NIHSS, mRS 0-3 (good functional outcome), and in-hospital mortality. Our findings were supported by some previous studies 7,23. The ENCHANTED trial 7 showed that major sICH occurred less frequently in the low-dose group compared to the standard-dose group (1.0% vs 2.1%, p=0.01). Moreover, the low-dose of alteplase was non-inferior to the standard dose in the ordinal analysis of mRS scores (unadjusted common OR 1.00; 95% CI 0.89 to 1.13; p=0.04 for non-inferiority). Data from a China national prospective stroke registry also revealed that in AIS patients with moderate severity of stroke, the lower-dose alteplase was associated with a lower risk of sICH and non-inferior performance in efficacy 23. In contrast, data from a randomized controlled trial and an observational study 15,24 did not show significant differences in the occurrence of sICH events between the low-dose and the standard-dose group. Whether the low-dose of alteplase may have a safer profile in treating AIS needs to further investigated. For example, evidence on optimal alteplase dosage in bridging thrombectomy after IVT remains scarce. A Korean prospective observational study 25 showed that AIS patients who received different doses of alteplase in the context of bridging therapy had no difference in functional outcome, whereas the risk of ICH was lower in the low-dose group compared to the standard-dose group (8% vs. 3%, p = 0.056). The optimal amount of thrombolysis alteplase for bridging thrombectomy in AIS is worth further investigating.

There are several explanations for the heterogeneous results regarding the relationship between alteplase dose and sICH risk of the abovementioned studies. First, different definitions of sICH were used, which may affect the diagnostic rate of sICH. In the present study, the ECASS-II criteria was used, which is a well-validated method to assess the sICH occurrence. Second, ethnic differences should not be underestimated. It has been reported that Asian populations have a higher risk of developing intracerebral bleeding events caused by intravenous thrombolysis 26. An observational study found that Japanese populations had a higher incidence of thrombolytic intracranial hemorrhage than Caucasians due to ethnic differences in clotting and fibrinolytic factor 27. Moreover, Asians were more likely to benefit from the low-dose alteplase 28.

Our study has some limitations. First, this is a retrospective observational study with a moderate sample size, inevitably introducing selection bias. Second, we only included AIS patients within 4.5 hours after onset. Our results therefore could not be generalized to those with stroke on awakening from sleep or unknown onset, or treatment in an extended time-window. Second, we only included East-Asian stroke population; therefore our findings are not generalizable to other ethnic stroke populations. Third, the low number of sICH events does not permit subgroup analysis stratified by age or stroke severity. Data from previous observational studies of East-Asian stroke populations supported a trend that low-dose tPA seemed to be safer in elderly patients 29,30. Fourth, we only assessed discharge functional outcomes; 90-day or longer-term follow-up of functional outcomes were not analyzed in the present study. Finally, our analysis was subject to casual post hoc grouping of alteplase doses, and thus, it is difficult to draw any conclusions based on the proper comparison.

## Conclusion

The current study showed that patients with increasing age, a higher baseline blood pressure, and previous stroke were at a higher odd of receiving a low dose of alteplase for patients with acute ischemic stroke within 4.5 hours of onset. The low-dose was related to a reduced risk of developing symptomatic intracranial hemorrhage. Further studies are needed to determine a safer profile for a low dose of alteplase in a selected stroke patient population.

## Figures and Tables

**Table 1:** Differences in baseline characteristics of patients in the standard-dose versus the low-dose alteplase

	Low-dose (n=96)	Standard-dose (n=410)	p-value
Female (n, %)	40 (41.7%)	138 (33.7%)	0.139
Age (y, median, IQR)	69 (63-79)	67 (58-74)	0.011
Current smoker (n, %)	29 (30.2%)	111 (27.1%)	0.537
Hypertension (n, %)	60 (62.5%)	282 (68.8%)	0.237
Hyperlipidemia (n, %)	37 (38.5%)	146 (35.6%)	0.590
Diabetes (n, %)	21 (21.9%)	91 (22.2%)	0.946
Atrial fibrillation (n, %)	33 (34.4%)	129 (31.5%)	0.582
Previous ischemic stroke (n, %)	20 (20.8%)	47 (11.5%)	0.015
Previous ICH (n, %)	0	0	NA
Chronic heart failure (n, %)	16 (16.7%)	79 (19.3%)	0.557
Baseline SBP (mmHg, median, IQR)	155 (132-168)	147 (134-160)	0.017
Baseline blood glucose (mmol/L, median, IQR)	6.8 (6.0-8.1)	6.8 (5.9-8.5)	0.788
Baseline blood glucose ≥ 10mmol/L (n, %)	14 (14.6%)	63 (15.4%)	0.848
Baseline hypertense MCA sign (n, %)*	12 (12.9%)	14 (3.5%)	<0.001
**Type of occluded vessel (n, %)**			0.233
Anterior circulation	80 (84.2%)	305 (74.6%)	
Posterior circulation	6 (6.3%)	39 (9.5%)	
Mixed	6 (6.3%)	36 (8.8%)	
Onset to thrombolysis time (min, median, IQR)^†^	180 (130-237)	170 (120-210)	0.184
Baseline NIHSS score (median, IQR)	7.5 (4-12)	6 (3-11)	0.022
sICH (n, %)	2 (2.1%)	32 (7.8%)	0.074
Fatal sICH (n, %)	0	3 (0.73%)	>0.999
Any ICH (n, %)	15 (15.6%)	74 (18.0%)	0.574
Discharge NIHSS	2 (1-5)	3 (1-6)	0.931
Discharge mRS 0-3 (n, %)	74 (77.1%)	296 (72.2%)	0.331
In-hospital mortality (n, %)	0	12 (2.9%)	0.135

**Abbreviations:** MCA, middle cerebral artery; NA, not applicable; NIHSS, national institute of health stroke scale; SBP, systolic blood pressure; sICH, symptomatic intracerebral hemorrhage; *Data available in 490 patients. ^†^Data available in 470 patients.

**Table 2:** Determinants for the utilization of the low-dose of alteplase

	Unadjusted (OR, 95%CI)	p-value	Age-sex-adjusted (OR, 95%CI)	p-value	Multivariable (OR, 95%CI)	p-value
Age (continuous)	1.03 (1.01-1.05)	0.004	/	/	1.02 (1.00-1.04)	0.042
Female sex	1.41 (0.89-2.22)	0.140	/	/	1.28 (0.80-2.06)	0.301
Previous ischemic stroke	2.03 (1.14-3.63)	0.016	1.93 (1.07-3.48)	0.029	2.01 (1.11-3.64)	0.021
Baseline NIHSS score (per-point increase)	1.04 (1.00-1.08)	0.031	1.03 (0.99-1.07)	0.097	1.04 (1.00-1.08)	0.057
Baseline SBP (per-10 mmHg increase)	1.13 (1.02 - 1.27)	0.026	1.00 (1.00 - 1.24)	0.071	1.12 (1.00 - 1.26)	0.049

**Abbreviations:** SBP, systolic blood pressure; OR, Odds ratio; CI, confidence interval; NIHSS, national institute of health stroke scale.

**Table 3:** Differences in baseline characteristics of patients who with and without sICH

	sICH (n = 34)	Non-sICH (n = 472)	p-value
Female (n, %)	15 (44.1%)	163 (34.5%)	0.258
Age (median, IQR), y	73 (68-80)	67 (58-74)	<0.001
Current smoker (n, %)	7 (20.6%)	133 (28.2%)	0.339
Hypertension (n, %)	25 (73.5%)	317 (67.2%)	0.444
Hyperlipidemia (n, %)	7 (20.6%)	176 (37.3%)	0.05
Diabetes (n, %)	12 (35.3%)	100 (21.2%)	0.056
Chronic heart failure (n, %)	9 (26.5%)	86 (18.2%)	0.234
Atrial fibrillation (n, %)	25 (73.5%)	137 (29.0%)	<0.001
Previous ischemic stroke (n, %)	4 (11.8%)	63 (13.3%)	0.999
Baseline SBP (mmHg, median, IQR)	142 (133-169)	149 (134-163)	0.573
Baseline blood glucose (mmol/L, median, IQR)	8.1 (6.5-11.6)	6.8 (5.9-8.3)	0.004
Baseline blood glucose ≥ 10mmol/L (n, %)	10 (29.4%)	67 (14.2%)	0.017
Baseline MCA hypertense sign (n, %)*	2 (6.3%)	24 (5.2%)	0.684
**Type of occluded vessel (n, %)**			0.203
Anterior	30 (88.2%)	355 (75.5%)	
Posterior	0	45 (9.6%)	
Mixed	3 (8.8%)	39 (8.3%)	
Onset to thrombolysis time (min)^†^	169 (123-210)	173 (120-210)	0.951
Baseline NIHSS score (median, IQR)	14 (9.5-17.5)	5 (3-11)	<0.001
Low-dose (n, %)	2 (5.9%)	94 (19.9%)	0.074

**Abbreviations:** MCA, middle cerebral artery; NIHSS, national institute of health stroke scale; SBP, systolic blood pressure; sICH, symptomatic intracerebral hemorrhage; *Data available in 490 patients; ^†^Data available in 470 patients.

**Table 4:** Association between the low-dose of alteplase and the risk of sICH

	Unadjusted (OR, 95%CI)	p-value	Age- sex- adjusted (OR, 95%CI)	p-value	Multivariable	p-value
Age (continuous)	1.07 (1.03-1.10)	0.001	/	/	1.06 (1.01-1.11)	0.016
Female sex	1.50 (0.74-3.02)	0.261	/	/	0.81 (0.35-1.85)	0.614
Hyperlipidemia	0.44 (0.19-1.02)	0.056	0.51 (0.22-1.22)	0.129	0.55 (0.21-1.40)	0.206
Diabetes	2.03 (0.97-4.24)	0.060	1.80 (0.85-3.82)	0.125	1.09 (0.40-2.96)	0.878
Atrial fibrillation	6.79 (3.09-14.93)	<0.001	5.35 (2.40-11.94)	<0.001	3.20 (1.33-7. 70)	0.009
Baseline blood glucose (>10mmol/L)	2.52 (1.15 - 5.50)	0.021	2.70 (1.21 - 6.02)	0.015	1.93 (0.64 - 5.83)	0.245
Baseline NIHSS score	1.19 (1.12-1.26)	<0.001	1.18 (1.11-1.25)	<0.001	1.16 (1.08-1.24)	<0.001
Low-dose	0.251 (0.06-1.07)	0.02	0.17 (0.04-0.76)	0.02	0.13 (0.03-0.62)	0.010

**Abbreviations:** OR, Odds ratio; CI, confidence interval; NIHSS, national institute of health stroke scale.

**Table 5:** Differences in baseline characteristics of low dose and standard dose using the propensity score-matching sample

	Low-dose (n = 88)	Standard-dose (n = 88)	p-value
Female, (n, %)	36 (40.9%)	26 (29.5%)	0.115
Age, (median, IQR),y	68 (60-78)	67 (61-72)	0.151
Hypertension, (n, %)	54 (61.4%)	67 (73.9%)	0.076
Hyperlipidemia, (n, %)	34 (38.6%)	42 (47.7%)	0.223
Diabetes, (n, %)	19 (21.6%)	23 (26.1%)	0.479
Chronic heart failure, (n, %)	14 (15.9%)	18 (20.5%)	0.434
Atrial fibrillation (n, %)	29 (33.0%)	33 (37.5%)	0.528
Previous ischemic stroke (n, %)	14 (15.9%)	8 (9.1%)	0.171
Baseline NIHSS score (median, IQR)	7 (4-12)	7 (4-12)	0.732
Hyperdense MCA sign (n, %)*	10 (11.4%)	6 (6.8%)	0.294
**Type of occluded vessel (n, %)**			0.640
Anterior	78 (84.1%)	71 (80.7%)	
Posterior	5 (5.7%)	4 (4.3%)	
Mixed	6 (6.8%)	11 (12.5%)	
Baseline SBP (mmHg) (median, IQR)	155 (132-165)	151 (136-167)	0.696
Baseline blood glucose (mmol/L)	6.7 (6.0-8.1)	7.0 (6.1-8.6)	0.352
Baseline blood glucose ≥ 10mmol/L (n, %)	14 (15.9%)	17 (54.8%)	0.553
Discharge NIHSS	2 (1-5)	2 (1-7)	0.732
Discharge mRS 0-3 (n, %)	61 (69.3%)	56 (63.6%)	0.425
sICH (n, %)	2 (2.3%)	10 (11.4%)	0.032
Any ICH (n, %)	14 (15.9%)	18 (20.5%)	0.434
Inhospital Mortality (n, %)	0 (0.0%)	3 (3.3)	0.246

**Abbreviations:** MCA, middle cerebral artery; NIHSS, national institute of health stroke scale; SBP, systolic blood pressure; sICH, symptomatic intracerebral hemorrhage; * Data available in 160 patients.

## Abbreviations

AIS: acute ischemic stroke
MCA: middle cerebral artery
mRS: modified Rankin scale
NIHSS: National Institutes of Health Stroke Scale
IQR: Interquartile range
IVT: intravenous thrombolysis
OR: odds ratios
CI: confidence interval
r-tPA: recombinant tissue-plasminogen activator
SBP: systolic blood pressure
SD: Standard deviation
